# Supraorbital Nerve Radiofrequency for Severe Neuralgia Caused by Herpes Zoster Ophthalmicus

**DOI:** 10.1155/2020/3191782

**Published:** 2020-09-25

**Authors:** Huan Zhang, Huadong Ni, Songlei Liu, Keyue Xie

**Affiliations:** ^1^Department of Anesthesiology, Xiasha Campus Sir Run Run Shaw Hospital, School of Medicine, Zhejiang University, Hangzhou, China; ^2^Department of Anesthesiology and Pain Medicine, First Affiliated Hospital of Jiaxing University, Jiaxing, China; ^3^Bengbu Medical College Graduate Department, Bengbu Medical College, Bengbu, China

## Abstract

**Background:**

Radiofrequency of the Gasserian ganglion can be used for ophthalmic herpetic neuralgia (OHN), but it is associated with complications. This study aimed to use the supraorbital nerve for computed tomography- (CT-) guided radiofrequency thermocoagulation to treat refractory OHN.

**Methods:**

This was a retrospective case series study of patients with simple or combined OHN treated at our hospital between 06/2012 and 06/2018. The numerical rating score (NRS), spontaneous pain, allodynia, gabapentin dosage, paracetamol/oxycodone dosage, patient global impression of change (PGIC) score, Barrow numbness score, postoperative 360-day recurrence rate, and complications were recorded before the operation and at 1, 30, 90, 180, and 360 days after the operation.

**Results:**

Compared with baseline, the NRS was decreased, and PGIC was increased at postoperative 1, 30, 90, 180, and 360 days, and the gabapentin and paracetamol oxycodone doses at postoperative 30, 90, 180, and 360 days were decreased (all *P* < 0.001). Compared with 1 day after the operation, numbness was decreased at 30, 90, 180, and 360 days after the operation (*P* < 0.001). Compared with baseline, the number of patients with allodynia at each time point after the operation was decreased (*P* < 0.001), but without a difference for spontaneous pain (*P*=0.407). No subjects showed drooping eyelid, corneal ulcers, eyeball damage, decreased vision, and other severe complications.

**Conclusion:**

CT-guided supraorbital nerve radiofrequency thermocoagulation for the treatment of OHN can effectively relieve pain and reduce the dose of analgesics, without any serious complication. This study suggests that this technique is feasible and applicable to clinical practice.

## 1. Introduction

Herpes zoster (HZ) is one of the clinical syndromes associated with the reactivation of latent varicella-zoster virus (VZV). HZ typically occurs years after primary VZV infection (varicella or chickenpox) [1–4]. The annual incidence of HZ is 3‰–5‰ [5]. HZ is most often characterized by a painful, unilateral vesicular skin eruption arising in a single dermatome (or sometimes adjacent dermatomes) [1–4]. The commonly involved sites are the thoracic, lumbar, cervical, and trigeminal dermatomes [1–4]. The skin eruptions are often preceded by a prodrome of pain or paresthesias occurring 1–5 days prior to the rash onset [1–4]. Advancing age is the major risk factor for HZ [6], but immunosuppressive diseases or therapies (including HIV infection) also increase the risk of HZ [1, 4]. Postherpetic neuralgia (PHN) (persistent pain >3 months beyond rash duration) is the most common complication of HZ, reported in 10%–50% of patients [1–4].

Cumulative trigeminal nerves (ophthalmic, maxillary, and mandibular nerves) can cause head and facial HZ, of which herpes zoster ophthalmicus (HZO) is the most common, which can account for 10%–20% of patients with HZ [7, 8]. HZO can cause ocular complications such as keratitis, but its most common symptom is herpetic neuralgia [9, 10], which seriously affects the quality of life of patients, especially the intractable pain associated with the long disease course. Furthermore, there is still a lack of effective treatment. Herpetic neuralgia is a type of neuropathic pain. The specific mechanism is unclear, but it is currently thought to be associated with the sensitization of the peripheral and central nervous system caused by VZV-induced neural inflammation or nerve injury [11, 12]. A good therapeutic effect can be achieved with the combined use of antivirus, analgesic drugs, and nerve block in the acute phase (herpes phase), but with the extension of the disease course to the subacute phase (≤1 month after herpes healing) or PHN phase (>1 month after herpes healing), neural sensitization is observed, and oral medication alone, normally, is unable to achieve satisfactory results. At this time, although local anesthetics and nerve block combination can have benefits, pain recurs after the drugs are metabolized, leading to a short duration of the treatment effect and occurrence of refractory herpetic neuralgia. Ophthalmic herpetic neuralgia in the trigeminal nerve is a challenge because of its special location and severe pain.

Radiofrequency (RF) is a reliable method for the treatment of neuropathic pain in patients who are refractory to conservative treatments. RF is divided into pulsed radiofrequency (neuromodulation) and radiofrequency thermocoagulation (neurolytic impairment). Pulsed radiofrequency has an unclear analgesia mechanism. Clinically, for the treatment of refractory herpetic neuralgia, a single-pulse radiofrequency has a short effective duration, and repeated operations are often necessary to prolong the curative effect, which will increase the patients' burden [13]. Meanwhile, radiofrequency thermocoagulation is to use high-temperature thermal degeneration to damage the nerves, blocking the upward transmission of pain signals to produce a reliable analgesic effect. It is applicable to selected patients with refractory herpetic neuralgia refractory to conservative treatment [14–18]. Currently, RF of the Gasserian ganglion is an effective method for the treatment of primary trigeminal neuralgia (TN) [14–17], but radiofrequency of the Gasserian ganglion carries a high risk in the treatment of ophthalmic herpetic neuralgia because it is located within the skull. It may cause serious complications such as corneal ulcer and vision loss after damage and decreased masticatory muscle strength, which often limits its application in ophthalmic herpetic neuralgia [14–17].

The aim of this retrospective study was to examine whether the use of another target, i.e., the supraorbital nerve, showed benefits for computed tomography- (CT-) guided radiofrequency thermocoagulation for the management of refractory ophthalmic herpetic neuralgia. The results could provide another option for the management of refractory ophthalmic herpetic neuralgia.

## 2. Materials and Methods

### 2.1. Study Design and Patients

This was a retrospective case series study of patients with simple or combined HZO treated at the pain department of our hospital between June 2012 and June 2018. The procedure was approved by the hospital in 2011 (#2011–037). The data were from a prospectively maintained database using Haitai 3.0 software. The data themselves were extracted from the charts after 2018. The study was approved by the ethics committee of our hospital. The need for individual consent was waived by the committee.

The inclusion criteria were as follows: (1) diagnosed with HZO [19], and the HZ lesions were distributed along the supraorbital nerve; (2) HZ was completely healed, as per clinical evaluation; (3) poor results after conservative treatment with drugs such as gabapentin (poor results: pain was not alleviated or nonsignificantly alleviated), or the side effects were intolerable; (4) numerical rating score (NRS) ≥6 points; and (5) completion of CT-guided radiofrequency thermocoagulation after unsuccessful therapeutic supraorbital nerve blockade with 2% lidocaine (2 ml) + compound betamethasone (1 ml) (refractory cases, i.e., effects disappearing over some time and NRS reaching ≥6 points again). The exclusion criterion was incomplete follow-up data.

### 2.2. Therapy

**Figure 1:**
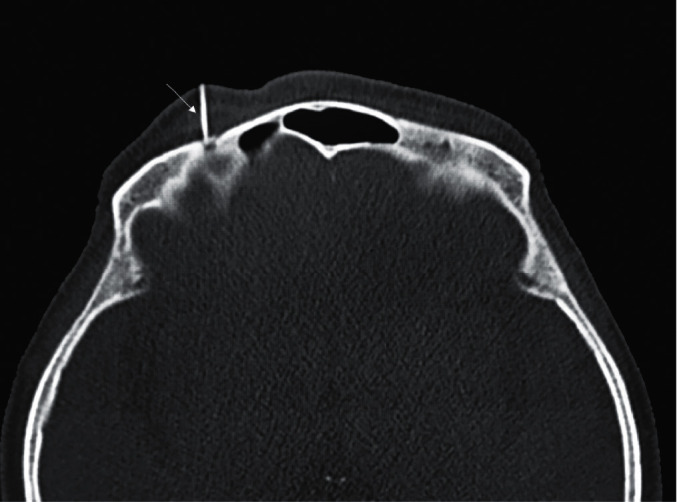
Computed tomography scan showing that the tip of the radiofrequency needle was located in the supraorbital foramen. The white line entering the skull is the radiofrequency needle (with white arrow pointing).

All operations were performed by the same surgeon, who was a chief physician who had been engaged in the diagnosis and treatment of pain for 20 years. The patient was placed supine on the operating table in the CT treatment room, comfortably fixed, with eyes closed. ECG was monitored routinely, and oxygen inhalation was given. The supraorbital foramen (notch) was identified using CT. When a complete supraorbital foramen (notch) could be seen, the CT layer was selected as the puncture layer, the puncture path was designed, and the puncture point was marked. After routine disinfection and draping, a radiofrequency puncture needle (length of 50 mm and inner diameter of 0.7 mm), exposed end of 2 mm (model: AN-N, specifications: 0.7 × 50–2 mm; Tuoren Medical Device Co., Ltd., Henan, China; registration number: CFDA 20153152153), was punctured vertically along the puncture path to the supraorbital orifice or the notch bottom (Figure 1) under the guidance of CT images. The radiofrequency instrument (PMG-230; Baylis Medical Company, Inc., Montreal, Canada) was connected, sensation (50 Hz) and motion (5 Hz) were tested, and the position of the needle was fine-tuned until the original pain area could be covered by both sensation and motion stimulations of <0.5 V. The treatment parameters were continuous standard radiofrequency, temperature of 95°C, and duration of 120 s [20]. The patients were given an intravenous injection of propofol (1–2 mg/kg, according to their age and general conditions) and then underwent radiofrequency thermocoagulation for 1-2 cycles. After the first cycle of radiofrequency was over, the patient was asked whether the original pain area was numb and whether the pain had disappeared. If the numb area does not completely cover the original pain area, the second cycle of radiofrequency is performed. After the operation, the sensation, pain, and corneal reflexes in the relevant areas were tested, and the patient was sent back to the ward. The entire operation lasted about 15–30 min.

### 2.3. Postoperative Pain Management

The patients continued to take oral gabapentin (Jiangsu Hengrui Medicine Co., Ltd., Jiangsu, China). The dose for patients with NRS 1–3 points was halved, while it could be doubled in patients with NRS 4–6 points; each increase or decrease lasted for more than 1 week. Patients with NRS >6 points were rescued with paracetamol oxycodone tablets (Specgx LLC, St Louis, MO, USA), no more than four tablets/day.

### 2.4. Data Collection

Before the operation and at 1, 30, 90, 180, and 360 days after the operation, the following data were recorded routinely: NRS, occurrence of spontaneous pain, occurrence of allodynia, gabapentin dosage, paracetamol/oxycodone dosage, the patient global impression of change (PGIC) score [21], the Barrow numbness score [22], postoperative 360-day recurrence rate (recurrence refers to the NRS equal to or greater than that before operation), and complications (such as subcutaneous swelling of the supraorbital area, eyelid droop, corneal ulcer, and eyeball damage). The recurrence and complications were obtained from the patient charts. PGIC scoring was 1: very bad; 2: aggravated severely; 3: aggravated slightly; 4: not changed; 5: improved slightly; 6: improved significantly; and 7: very good. The Barrow numbness score was 1: no facial numbness; 2: facial numbness, not bothering; 3: facial numbness, bothering; and 4: facial numbness, very bothering.

### 2.5. Statistical Analysis

Statistical analyses were performed using SPSS 16.0 (SPSS Inc., Chicago, IL, USA). Continuous data were first tested with the Kolmogorov–Smirnov normality test. Data in line with a normal distribution were expressed as means ± standard deviations, and comparison between pre- and posttreatment data was conducted using repeated-measures ANOVA and the post hoc paired *t*-test. Data with a skewed distribution were expressed as medians (P_25_, P_75_); comparison between pretreatment and posttreatment data was conducted using the Friedman rank-sum test, and multiple comparisons were made using the Wilcoxon rank-sum test. Categorical data were analyzed using the Pearson chi-square test. Two-sided *P* values <0.05 were considered statistically significant.

## 3. Results

### 3.1. Patients

Thirty-six patients among eighty patients with HZO completed the radiofrequency thermocoagulation intervention. Five patients were lost to follow-up, and 31 patients were finally analyzed in the study. Their characteristics are listed in Table 1. There were 19 males. The mean age was 73.6 ± 9.9 years.

### 3.2. Changes in Pain and Numbness Indicators

The last follow-up was on September 1, 2019. The NRS, gabapentin dosage, paracetamol/oxycodone dosage, PGIC before and after the operation, and postoperative numbness are shown in Table 2. Compared with before operation, the NRS was decreased, and PGIC was increased at postoperative 1, 30, 90, 180, and 360 days, and the gabapentin and paracetamol oxycodone doses at postoperative 30, 90, 180, and 360 days were decreased (*χ*^2^=80.37, *χ*^2^=55.53, *χ*^2^=38.00, and *χ*^2^=47.32; all *P* < 0.001). The differences in gabapentin and paracetamol oxycodone doses at postoperative 1 day were not statistically significant (*Z*=−0.56 and *Z*=−0.54; both *P* > 0.05). Compared with 1 day after the operation, numbness was decreased at 30, 90, 180, and 360 days after the operation (*χ*^2^=80.00, *P* < 0.001).

The number of patients with allodynia and spontaneous pain is shown in Table 3. Compared with before operation, the number of patients with allodynia at each time point after the operation was decreased (*χ*^2^=46.25, *P* < 0.001), while the number of patients with spontaneous pain at each time point after the operation did not show a significant difference (*χ*^2^=5.07, *P*=0.407). In addition, there were no significant differences between the number of patients with allodynia and the number of patients with spontaneous pain before operation (*χ*^2^=0.52, *P*=0.472), but the number of patients with spontaneous pain at each time point after the operation was higher than the number of patients with allodynia (*χ*^2^=37.71, *χ*^2^=28.93, *χ*^2^=22.03, *χ*^2^=18.08, and *χ*^2^=9.23; all *P* < 0.01).

### 3.3. Complications

At 360 days after the operation, four patients (13%) had recurrences, but they were treated at other hospitals, and the details are unknown. Twenty-nine (94%) patients had varying degrees of subcutaneous swelling at the supraorbital area within 1 week of surgery, but no patients showed drooping eyelid, corneal ulcers, eyeball damage, decreased vision, and other severe complications. After 1 week without special treatment, they returned to normal on their own.

## 4. Discussion

Radiofrequency of the Gasserian ganglion can be used for ophthalmic herpetic neuralgia, but it is associated with risks and complications [14–17]. This study aimed to use the supraorbital nerve for CT-guided radiofrequency thermocoagulation to treat refractory ophthalmic herpetic neuralgia. The results suggest that CT-guided supraorbital nerve radiofrequency thermocoagulation for the treatment of ophthalmic herpetic neuralgia can effectively relieve pain and reduce the dose of analgesics, without any serious complication. CT can confirm whether the needle tip enters the supraorbital hole, which is the biggest advantage of CT guidance compared to B-mode ultrasound guidance (which has limited application in the skull if no acoustic window is possible) or blind procedure. During the operation, CT can clearly guide the operator on the distance and the relative position between the puncture needle and the target point, i.e., the supraorbital hole, while the guidance of B-ultrasound is relatively vague and not precise enough. CT can be accurate to the range of 1 mm, while B-ultrasound cannot reach this accuracy and can only roughly guide the approximate position and direction. Once entering the supraorbital hole, the puncture needle will be stuck into the hole, and the position will naturally be fixed. This case series suggests that this technique is feasible and applicable to clinical practice.

The Gasserian ganglion, which is a concentration site of primary sensory neuron bodies in the head and face, is the ideal target for radiofrequency thermocoagulation in the treatment of refractory ophthalmic herpetic neuralgia [14–17]. Radiofrequency of the Gasserian ganglion was first proposed by Hartel [23] about 100 years ago, who performed puncture to the Gasserian ganglion through the oval foramen under X-ray guidance. It was a significant milestone at the time but was limited to the medical conditions at that time, and now imaging of CT has far exceeded X-ray, and more accurate and clear images are obtained. Nowadays, the treatment of trigeminal neuralgia using radiofrequency of the Gasserian ganglion is no longer necessary. Instead, CT can complete highly selective branch radiofrequency treatment of the trigeminal neuralgia, that is, V1 is in the supraorbital hole, V2 in the foramen rotundum, and V3 in the oval foramen. The advantage of such selective radiofrequency is that it does not need to enter the cranium (the Gasserian ganglion is in the cranium), it is safer (absolutely no intracranial infection, intracranial hemorrhage, and damage to other nondiseased trigeminal nerve branches), and it is more effective (in the past, radiofrequency of the Gasserian ganglion required repeated electrophysiological tests in the Gasserian ganglion to try to induce sensory and movement corresponding to the innervation area of the diseased branch, that is, to replicate the original pain. This process is often not easy, especially for V1 and V2, which is time-consuming and laborious, and the patient has to endure severe pain and cannot receive anesthesia. Therefore, the radiofrequency of the Gasserian ganglion is a technology that has been or will be abandoned. Indeed, the ophthalmic nerve root is located deep in the Gasserian ganglion, resulting in difficult puncture, high risk, and difficulty in reaching the target, and radiofrequency thermocoagulation of the ophthalmic nerve root in the Gasserian ganglion is prone to cause corneal ulcers [14]. Therefore, targeting the Gasserian ganglion might not be the best approach. The efficacy of radiofrequency thermocoagulation of the trigeminal nerve root was reported for the treatment of PHN in the head and face (eight cases of the ophthalmic nerve and one case of the mandibular nerve), among whom eight patients achieved pain relief after the operation, but one patient had keratitis, and two had corneal ulcers [24].

The ophthalmic nerve erupts from the skull via the supraorbital fissure and is immediately branched into the lacrimal, nasociliary, and frontal nerves [25]. The nasociliary nerve dominates and nourishes the cornea and other parts [26]. The frontal nerve travels on the deep levator muscle and branches into the supraorbital and supratrochlear nerves at the orbital apex, which separately penetrate through the supraorbital foramen (notch) and the supratrochlear notch to dominate periorbital and frontal apex skin [27]. Both supraorbital and supratrochlear nerves are responsible for supraorbital herpes, of which the area dominated by the supraorbital nerve is more commonly found. It was reported that keratitis and corneal ulcers in HZO are closely related to nasociliary nerve injury [28]. Therefore, radiofrequency thermocoagulation of peripheral nerves (supraorbital nerves) can be far away from the nasociliary nerve to reduce the probability of the injury, and thus, the occurrence of postoperative corneal ulcers can be reduced compared with radiofrequency thermocoagulation of the Gasserian ganglion. Based on this, shifting the target outward and attempting to treat refractory ophthalmic herpetic neuralgia with radiofrequency thermocoagulation of the supraorbital nerve under CT guidance were tried.

Among the 31 patients, the pain score decreased significantly after radiofrequency thermocoagulation, and the relief of allodynia was significantly better than that of spontaneous pain. Spontaneous pain is considered to be caused by central sensitization [29]. This type of pain cannot be resolved by radiofrequency of the orbital foramen. Peripheral nerve radiofrequency can only relieve the allodynia caused by peripheral sensitization [30]. The allodynia disappeared in most patients after operation, and the spontaneous pain was alleviated, although it could not be eliminated. Different effects on allodynia and spontaneous pain might be associated with different mechanisms. Allodynia is caused because of the A_*β*_ fibers sprout from the deep lamellar to lamellar I and lamellar II in the spinal dorsal horn after nerve injury and occupies the upward pain pathway of the A_*β*_ and C fibers [31, 32], which belongs to stimulus-dependent pain. It can cause pain by touching the sensory nerve endings in the skin. Destroying the peripheral nerve fibers can effectively block the tactile upward pathway in the nerve endings and eliminate pain. Meanwhile, spontaneous pain is caused by abnormal discharge after nerve sensitization [33]. Since the abnormal discharge point is uncertain, it is a kind of non-stimulus-dependent pain and only refers to the damage to peripheral nerve fibers. The pain caused by an abnormal discharge in the sensory conduction upstream cannot be completely eliminated. Hence, radiofrequency thermocoagulation of the supraorbital nerve may be more suitable for patients who mainly suffer from allodynia rather than spontaneous pain, but this will require further research.

With the relief of pain after radiofrequency, the doses of analgesics such as gabapentin and paracetamol oxycodone after the operation could be decreased accordingly. In the present study, there was no significant change in the doses at postoperative 1 day, which may be related to pain and discomfort due to subepithelial swelling in the supraorbital area after thermocoagulation. Meanwhile, PGIC, as an indicator that effectively reflects the therapeutic effect, was improved postoperatively compared with baseline (after nerve block), indicating that the treatment is effective.

Numbness is an unpleasant sensory experience and is also a cause for the low acceptance of nerve impairment methods [34]. In the present study, all patients had different degrees of numbness after radiofrequency thermocoagulation of the supraorbital nerve, but due to the high selectivity of the supraorbital nerve, numbness was limited to the forehead and the apex and did not affect the motor functions. During follow-up, it was found that postoperative numbness gradually decreased, and the number of patients with allodynia gradually increased with time. There were four recurrent cases, with a recurrence rate of up to 13%. In contrast to the Gasserian ganglion, which is composed of the neuronal soma, the supraorbital nerve is composed of nerve fibers. It has been proved that nerve fibers can regenerate to a certain extent after injury [35]. Therefore, after radiofrequency thermocoagulation, the sensory neuronal soma may not be damaged, which can promote nerve fiber regeneration, gradually recovering the nerve conduction function, reduce numbness, and increase the possibility of postoperative pain recurrence. Thus, radiofrequency thermocoagulation may have a higher recurrence rate compared with other methods, but further comparison is needed. An important point that still needs to be explored about radiofrequency ablation of the supraorbital nerve is how to prolong the effective time and reduce the recurrence of allodynia after the operation. Most patients display varying degrees of subcutaneous swelling after RF ablation because the supraorbital blood vessels travel along with the ablated nerves, and the subcutaneous soft tissues in the peripheral area are loose, allowing for edema. Nevertheless, this edema can be alleviated within 1-2 weeks after symptomatic treatment, without any adverse effect. During the present study, no serious adverse reactions such as eyelid droop, corneal ulcers, and eyeball injury occurred.

This retrospective study has some limitations. The sample size was small. There was no control group. In addition, the patients combined with HZ in the supratrochlear nerve were excluded, but some cases might have been missed since there is a somatic overlap between both nerves. Therefore, further research and observations are needed.

## 5. Conclusion

In conclusion, radiofrequency thermocoagulation of the supraorbital nerve is a feasible method for treating ophthalmic herpetic neuralgia, without serious complications. This could be a suitable method for the management of ophthalmic herpetic neuralgia.

## Figures and Tables

**Table 1:** Characteristics of the patients.

Parameters		Mean ± SD or *N* (%)
Age (years)		73.6 ± 9.9
Sex, *n* (%)	Male	19 (61%)
Location, *n* (%)	Left	14 (45%)
Affected nerves, *n* (%)	I	26 (84%)
	I + II	4 (13%)
	I + II + III	1 (3%)
Disease course (after herpes healed), *n* (%)	≤1 month	12 (39%)
	>1 month	19 (61%)
Combined chronic diseases, *n* (%)	Hypertension	27 (87%)
	Diabetes	10 (32%)
Involved facial nerve, *n* (%)		1 (3%)
Frequency of preoperative nerve blocks		7.4 ± 1.5

**Table 2:** Comparison of NRS, gabapentin (g), paracetamol oxycodone (mg), PGIC, and numbness before and after PRF (median (P_25_, P_75_)).

Parameters	Before intervention	1 day after intervention	30 days after intervention	90 days after intervention	180 days after intervention	360 days after intervention	*P*
NRS	6.0 (5.0, 6.5)	2.0 (2.0, 3.0)^*∗*^	2.0 (1.0, 2.0)^*∗*^	2.0 (1.0, 3.0)^*∗*^	2.0 (1.0, 3.0)^*∗*^	2.0 (1.0, 3.0)^*∗*^	<0.001
Gabapentin (g)	0.6 (0.6, 0.9)	0.6 (0.6, 0.9)	0.6 (0.0, 0.9)^*∗*^	0.6 (0.0, 0.9)^*∗*^	0.6 (0.0, 0.9)^*∗*^	0.0 (0.0, 0.9)^*∗*^	<0.001
Paracetamol oxycodone (mg)	0.0 (0.0, 5.0)	0.0 (0.0, 5.0)	0.0 (0.0, 0.0)^*∗*^	0.0 (0.0, 0.0)^*∗*^	0.0 (0.0, 0.0)^*∗*^	0.0 (0.0, 0.0)^*∗*^	<0.001
PGIC	4.0 (4.0, 5.0)	5.0 (5.0, 5.0)^*∗*^	5.0 (5.0, 5.0)^*∗*^	5.0 (5.0, 5.0)^*∗*^	5.0 (5.0, 6.0)^*∗*^	5.0 (5.0, 6.0)^*∗*^	<0.001
Numbness		3.0 (3.0, 4.0)	3.0 (2.0, 3.0)^#^	2.0 (2.0, 3.0)^#^	2.0 (2.0, 3.0)^#^	2.0 (2.0, 3.0)^#^	<0.001

^*∗*^ *P* < 0.05 vs. preoperation. ^#^*P* < .05 vs. 1 month after the operation. NRS: numerical rating score; PGIC: patient general impression of change score.

**Table 3:** Comparison of the number of patients with allodynia and spontaneous pain.

Parameters	Before intervention	1 day after intervention	30 days after intervention	90 days after intervention	180 days after intervention	360 days after intervention	*P*
Allodynia, *n* (%)	29 (94%)	6 (19%)^*∗*^	7 (23%)^*∗*^	10 (32%)^*∗*^	14 (45%)^*∗*^	19 (61%)^*∗*^	<0.001
Spontaneous pain, *n* (%)	31 (100%)	29 (94%)^#^	28 (90%)^#^	28 (90%)^#^	29 (94%)^#^	29 (94%)^#^	0.407
*P*	0.472	<0.001	<0.001	<0.001	<0.001	0.002	

^*∗*^ *P* < 0.05 vs. preoperation. ^#^*P* < 0.05 vs. 1 month after the operation.

## Data Availability

The datasets used and/or analyzed during the current study are available from the corresponding author on reasonable request.

## References

[1] Schmader K. (2018). Herpes zoster. *Annals of Internal Medicine*.

[2] Saguil A., Kane S., Mercado M., Lauters R. (2017). Herpes zoster and postherpetic neuralgia: prevention and management. *American Family Physician*.

[3] Sampathkumar P., Drage L. A., Martin D. P. (2009). Herpes zoster (shingles) and postherpetic neuralgia. *Mayo Clinic Proceedings*.

[4] Cohen J. I. (2013). Herpes zoster. *New England Journal of Medicine*.

[5] Kawai K., Gebremeskel B. G., Acosta C. J. (2014). Systematic review of incidence and complications of herpes zoster: towards a global perspective. *BMJ Open*.

[6] Johnson B. H., Palmer L., Gatwood J. (2015). Annual incidence rates of herpes zoster among an immunocompetent population in the United States. *BMC Infectious Diseases*.

[7] Liesegang T. (2008). Herpes zoster OphthalmicusNatural history, risk factors, clinical presentation, and morbidity. *Ophthalmology*.

[8] Catron T., Hern H. G. (2008). Herpes zoster ophthalmicus. *The Western Journal of Emergency Medicine*.

[9] Ting D. S. J., Ghosh N., Ghosh S. (2019). Herpes zoster ophthalmicus. *BMJ*.

[10] Freund P. R., Chen S. H. (2018). Herpes zoster ophthalmicus. *Canadian Medical Association Journal*.

[11] Mallick-Searle T., Snodgrass B., Brant J. (2016). Postherpetic neuralgia: epidemiology, pathophysiology, and pain management pharmacology. *Journal of Multidisciplinary Healthcare*.

[12] Costigan M., Scholz J., Woolf C. J. (2009). Neuropathic pain: a maladaptive response of the nervous system to damage. *Annual Review of Neuroscience*.

[13] Kim K., Jo D., Kim E. (2017). Pulsed radiofrequency to the dorsal root ganglion in acute herpes zoster and postherpetic neuralgia. *Pain Physician*.

[14] Guo J., Dong X., Zhao X. (2016). Treatment of trigeminal neuralgia by radiofrequency of the Gasserian ganglion. *Reviews in the Neurosciences*.

[15] Telischak N. A., Heit J. J., Campos L. W., Choudhri O. A., Do H. M., Qian X. (2018). Fluoroscopic C-arm and CT-guided selective radiofrequency ablation for trigeminal and glossopharyngeal facial pain syndromes. *Pain Medicine*.

[16] Choudhri Y., Hong T., Li H., Yao P., Zhao G. (2019). Efficacy of CT guided pulsed radiofrequency treatment for trigeminal postherpetic neuralgia. *Frontiers in Neuroscience*.

[17] Shrestha M., Chen A. (2018). Modalities in managing postherpetic neuralgia. *The Korean Journal of Pain*.

[18] Wan C. F., Liu Y., Dong D. S. (2016). Bipolar high-voltage, long-duration pulsed radiofrequency improves pain relief in postherpetic neuralgia. *Pain Physician*.

[19] Kong C. L., Thompson R. R., Porco T. C., Kim E., Acharya N. R. (2020). Incidence rate of herpes zoster ophthalmicus: a retrospective cohort study from 1994 through 2018. *Ophthalmology*.

[20] Ding Y., Li H., Hong T., Zhao R., Yao P., Zhao G. (2019). Efficacy and safety of computed tomography-guided pulsed radiofrequency modulation of thoracic dorsal root ganglion on herpes zoster neuralgia. *Neuromodulation: Technology at the Neural Interface*.

[21] Zhao D., Bhatia A., McParland A. (2020). Evaluating the impact of gabapentinoids on sleep health in patients with chronic neuropathic pain: a systematic review and meta-analysis. *Pain*.

[22] Ran B., Wei J., Zhong Q. (2019). Long-term follow-up of patients treated with percutaneous radiofrequency thermocoagulation via the foramen rotundum for isolated maxillary nerve idiopathic trigeminal neuralgia. *Pain Medicine*.

[23] Hartel F. (1914). Uber die intracranielle injektionsbehandlung der trigeminusneuralgie. *Medizinische Klinik*.

[24] Teixeira M. J., Siqueira S. R. D. T., Almeida G. M. (2006). Percutaneous radiofrequency rhizotomy and neurovascular decompression of the trigeminal nerve for the treatment of facial pain. *Arquivos de Neuro-Psiquiatria*.

[25] Huff T., Daly D. T. (2020). *Neuroanatomy, Cranial Nerve 5 (Trigeminal)*.

[26] Szent-Ivanyi J., Hassan A. S., Teimory M. (2014). Herpes zoster ophthalmicus: is the globe involved?. *BMJ Case Reports*.

[27] Myckatyn T. M., Mackinnon S. E. (2004). A review of facial nerve anatomy. *Seminars in Plastic Surgery*.

[28] Li J. Y. (2018). Herpes zoster ophthalmicus. *Current Opinion in Ophthalmology*.

[29] Woolf C. J. (2011). Central sensitization: implications for the diagnosis and treatment of pain. *Pain*.

[30] Colloca L., Ludman T., Bouhassira D. (2017). Neuropathic pain. *Nature Reviews Disease Primers*.

[31] Woolf C. J., Shortland P., Coggeshall R. E. (1992). Peripheral nerve injury triggers central sprouting of myelinated afferents. *Nature*.

[32] Wu C.-H., Yuan X.-C., Gao F. H.-P. (2016). Netrin-1 contributes to myelinated afferent fiber sprouting and neuropathic pain. *Molecular Neurobiology*.

[33] Li S. R., Luo S., Henry M. A. (2012). The role of sodium channels in chronic pain. *Muscle & Nerve*.

[34] Liu G., Du Y., Wang X., Ren Y. (2019). Efficacy and safety of repeated percutaneous radiofrequency thermocoagulation for recurrent trigeminal neuralgia. *Frontiers in Neurology*.

[35] Scheib J., Höke A. (2013). Advances in peripheral nerve regeneration. *Nature Reviews Neurology*.

